# A Comparative Study between Ultrasound Cycloplasty and Cyclocryotherapy for the Treatment of Neovascular Glaucoma

**DOI:** 10.1155/2020/4016536

**Published:** 2020-01-22

**Authors:** Wang Ruixue, Wang Tao, Li Ning

**Affiliations:** Department of Ophthalmology, The First Affiliated Hospital of Anhui Medical University, Hefei, Anhui Province, China

## Abstract

**Purpose:**

To compare the clinical efficacy, safety, and histological effect between ultrasound cycloplasty (UCP) and cyclocryotherapy in the treatment of neovascular glaucoma.

**Methods:**

Two groups of neovascular glaucoma patients who underwent two types of treatment, respectively, namely, 26 patients treated by UCP and 23 by cyclocryotherapy, were treated and observed during the clinical study for six months. The primary outcome was evaluated by the surgical success, which was defined as the intraocular pressure (IOP) reduction of greater than or equal to 20% from the baseline and the IOP value of greater than 5 mmHg at the last follow-up. The secondary outcome referred to pain relief, complications, and the mean of the IOP at each follow-up. In the animal experiment, 18 New Zealand rabbits were divided into two groups and treated by UCP and cyclocryotherapy, respectively. The changes in the tissues and in the expression of matrix metalloproteinase-1 (MMP-1) were observed immediately.

**Results:**

The mean IOP baseline for the UCP and cyclocryotherapy groups was 54.6 ± 9.7 mmHg and 53.3 ± 11.7 mmHg, respectively. After six months of follow-up, the IOP value decreased to 30.3 ± 9.4 mmHg for the patients treated by UCP and to 30.4 ± 9.1 mmHg for those treated by cyclocryotherapy. The two groups achieved a satisfying success rate in the treatment of neovascular glaucoma of up to 70% at least. Vision impairment was observed in some patients treated with cyclocryotherapy, and these patients suffered from more complications and less pain relief than the patients who were treated with UCP. The histological study showed that the ciliary body was completely destroyed after cyclocryotherapy and that MMP-1 was found only in the ciliary muscle. After the UCP treatment, MMP-1 could still be found in the ciliary body, and only the double-layer epithelial cells presented with coagulative necrosis.

**Conclusion:**

The UCP treatment and cyclocryotherapy both showed good efficacy in significantly reducing the IOP. However, the UCP treatment was safer with less postoperative complications and adverse effects. Thus, the overall treatment effect of the UCP was more efficient than that of cyclocryotherapy.

## 1. Introduction

Neovascular glaucoma (NVG) is a type of refractory glaucoma associated with a complex etiology and a significant risk of blindness. It is characterized by the occurrence of new vessels in the iris and angle that is difficult to deal with [1]. The angle of the anterior chamber is closed because of the contraction of the intraocular fibrovascular membranes in the eye, thus impeding the outflow of the aqueous humor and causing an uncontrollable high intraocular pressure (IOP). Patients are not only at high risk of blindness but also suffer from severe eye pain. Moreover, it is difficult to treat [2, 3]. The method to prevent the progressive loss of vision has not yet been found until now, and reducing the IOP remains the mainstay of glaucoma treatment.

In most patients with advanced NVG, hypotensive medication has been unable to control the IOP and surgery has become the only possible treatment option [4]. Many surgical methods can be used to reduce the IOP in NVG. Surgeries such as trabeculectomy, drainage valve implantation, cyclocryotherapy, and diode laser ring photocoagulation, among others, are commonly performed in clinical practice. Among these procedures, cyclocryotherapy has been used to treat NVG since the 1960s. It not only can reduce the production of aqueous humors but also can increase its outflow. The efficacy of cyclocryotherapy in reducing the IOP is widely recognized. However, it still has some flaws. For example, during the treatment, the dosage is difficult to control, or the positioning is not precise enough. Moreover, complications at different degrees are common after surgery [5, 6].

Ultrasound cycloplasty (UCP), as a new noninvasive treatment of glaucoma that uses high-intensity focused ultrasound to coagulate the ciliary body, reduces the IOP in a gentle and comfortable way through a dual impact on the aqueous humor dynamics [7–10].

To understand the efficacy of the two procedures, this study compared the UCP treatment and cyclocryotherapy in terms of the IOP reduction, pain relief, and possible complications.

## 2. Materials and Methods

### 2.1. Patients

This is a prospective study. Two groups of patients were treated, in the cyclocryotherapy group, 23 eyes of 23 patients were treated from September 2017 to October 2018. And in the UCP group, 26 eyes of 26 patients were treated from November 2018 to June 2019. The patients in the UCP and cyclocryotherapy groups, aged 25–80 years and 23–80 years, respectively, were all adults.

This study was conducted in accordance with the principles of the Declaration of Helsinki and ISO 14155 standard and was approved by the local institutional review board. Written content was obtained from all the enrolled patients.

### 2.2. Inclusion Criteria

(1) Patients diagnosed with NVG; (2) hypotensive medication is insufficient to control the IOP; (3) IOP greater than or equal to 20 mmHg; (4) age greater than 18 years old and less than 90 years old; (5) patients who signed the informed consent and who were able to complete all postoperative follow-up visits; and (6) type and amount of ocular hypotensive medication should remain the same before and after treatment.

### 2.3. Exclusion Criteria

(1) Eye infection in any eye two weeks before treatment; (2) any medical, treatment history, and systemic disease that can affect the evaluation of the treatment efficacy; (3) pregnant or lactating women; (4) patients who need to conduct other procedures at the same time; and (5) patients who underwent other treatments for reducing the IOP within six months after the UCP or cyclocryotherapy.

### 2.4. Preoperative Examination

Routine eye examinations, such as uncorrected visual acuity, photography of the anterior segment, IOP measurement, and pain assessment, were performed before treatment. For IOP measurement, we used the Goldman applanation tonometer. Ultrasound biomicroscopy (UBM), axial length, and white-to-white distance measurements were performed before the UCP treatment to define the probe size to be used for each patient.

### 2.5. Treatment Procedure

Both anesthesia and treatment were performed by the same experienced ophthalmologist. Retrobulbar anesthesia was applied in all patients, and tobramycin dexamethasone eye drops were prescribed one month after treatment.

#### 2.5.1. UCP

The procedure was performed using the medical device EyeOP1 (Eye Tech Care, France). The device consists of a command module and a disposable therapy device including a positioning cone and a treatment probe. The treatment probe uses six transducers in a circle to locate the ciliary process with sub-millimeter accuracy and a strict temperature control [4, 11]. The operator can determine the treatment dosage precisely according to the patients' condition. The probe is available in three diameters (11, 12, and 13 mm). UBM is used to match the suitable probe size for the patients before treatment. The specific steps are as follows. (1) With the patient lying in a supine position and after starting the device, fix and align the positioning cone on the ocular surface of the patient. (2) Check the negative pressure, ensure that there is no leak and then put the treatment probe into the positioning cone. Fill the cone with saline solution. Step on the pedal and start the treatment. (3) After the treatment is completed, remove the positioning cone and treatment probe. Patients should stay in the hospital for observation for 2 h.

#### 2.5.2. Cyclocryotherapy

A cryoprobe with a 25 mm diameter is applied. It freezes a 2 mm area behind the corneal limb at 180°. Press the probe against the sclera until a 3-4 mm frozen zone is formed and then start the timing. The probe freezes each site at −80°C for 40–60 s. Remove the probe when the temperature returns to normal. Patients need to be hospitalized for a three-day observation.

### 2.6. Postoperative Follow-Up

Follow-up visits were scheduled on day 1, week 1, month 1, month 3, and month 6 after treatment. Eye examinations such as uncorrected visual acuity, photography of the anterior segment, IOP measurement, and pain and complication assessment were performed.

### 2.7. Outcome Measures

The qualified success criteria were defined as an IOP reduction of greater than or equal to 20% compared with the baseline value and an IOP greater than 5 mmHg at the last follow-up visit.

#### 2.7.1. Pain Assessment Scale

To make the assessment easy for patients to understand, a pain scale of 0–10 was used in this study. The patients evaluated their pain level by themselves (score of 0 = no pain and score of 10 = unbearable pain).

### 2.8. Histology

The high IOP model was induced by the compound carbomer in 18 New Zealand rabbits, which were divided into the UCP treatment group and the cyclocryotherapy group. General anesthesia was induced by the intravenous injection of 10% chloralhydrate (3.5 mg/kg) into the ear vein. Each New Zealand rabbit was treated by UCP or cyclocryotherapy in one eye, and the other eye was used as a negative control. The rabbits were executed immediately after the treatment. The two eyeballs were removed and preserved in a 10% formalin solution. Twenty-four hours later, the eyeballs were embedded in paraffin to form a 5 *μ*m-thick paraffin section. The histological patterns were observed under a light microscope after HE staining.

### 2.9. Immunohistochemistry of Matrix Metalloproteinase-1 (MMP-1)

After dewaxing, the paraffin sections were high-pressure treated and incubated in a 0.3% hydrogen peroxide solution for 10 min at room temperature. Then, the paraffin sections were rinsed with PBS and blocked in goat serum for 10 min at room temperature. We discarded the goat serum blocking reagent solution and then added the diluted primary antibody (MMP-1 1 : 100) to the paraffin sections and incubated at 4°C overnight. After washing the paraffin sections with PBS, we added a secondary antibody to the paraffin sections incubated at room temperature for 1 h. We washed the paraffin sections again and added HRP-conjugated streptavidin. The paraffin sections were washed again with PBS for 20 min. After the DAB coloration, counterstaining, and mounting, the paraffin sections were observed under a microscope. PBS was used as the negative control instead of the primary antibody.

### 2.10. Statistical Analysis

Data were analyzed by SPSS23.0 statistical software (IBM, USA). Descriptive statistics was used to report the demographic and ocular baseline characteristics. The Wilcoxon rank sum test, Fisher's exact test, and chi-square test were used for the demographic analysis. For the continuous variables, the nonparametric Mann–Whitney test was performed to detect the differences among the groups. Statistical significance was set to *P* < 0.05.

## 3. Results

### 3.1. Patient Characteristics

Surgery was performed smoothly in all patients. The differences in characteristics between the two groups were not statistically significant. They are shown in Table 1 for details.

### 3.2. Intraocular Pressure

In the UCP group, the mean IOP was compared with the baseline on day 1, week 1, month 1, month 3, and month 6 after treatment, and the difference was statistically significant (*P* < 0.05). In the cyclocryotherapy group, the mean IOP was compared with the baseline on day 1, week 1, month 1, month 3, and month 6 after treatment, and the difference was also statistically significant (*P* < 0.05).

The IOP reduction in all patients at each follow-up visit is shown in Table 2.

**Figure 1:**
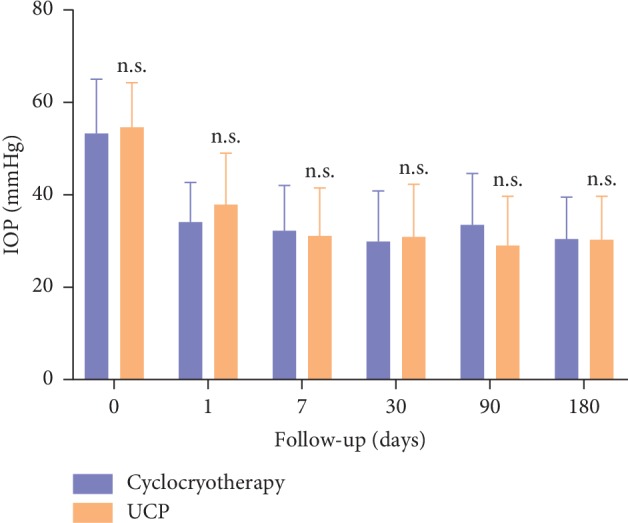
The mean IOP of all patients. Note: ^*∗*^*P* < 0.001; ^*∗∗*^*P* < 0.01; ^*∗∗∗*^*P* < 0.05; n.s. no statistical significance.

The mean IOP of all patients is illustrated in Figure 1. At each follow-up, no statistically significant difference was found in the IOP between the two groups.

### 3.3. Pain Assessment

Six months later, the number of patients without pain accounted for 90% of the final follow-up in the UCP group and 73% in the cyclocryotherapy group.

The pain assessment of patients in the two groups is presented in Table 3.

### 3.4. Complications

Complications occurred in both groups. The difference in the postoperative visual acuity was the most significant. The visual acuity of the patients treated by UCP remained the same before and after treatment. Conversely, visual acuity was impaired in some patients treated by cyclocryotherapy, as shown in Table 4.

The other complications that occurred during the follow-up visits are shown in Table 5.

Fewer complications were found after the UCP treatment than after cyclocryotherapy. Postoperative complications occurred in 8 cases after the UCP treatment and in 23 cases after cyclocryotherapy.

### 3.5. Histological Effect

In the UCP treatment group, the coagulated parts of the ciliary body were distributed periodically and evenly in the sectors, except in the nasal and temporal regions. The high-intensity focused ultrasound mainly targeted the ciliary process and produced a thermal effect to coagulate the epithelial cells that secrete the aqueous humor. In the affected area, coagulative necrosis and inflammatory reactions caused interstitial edema and vasodilatation in the ciliary process. However, the basal region of the ciliary process and the rest of the ciliary body remained normal. The double-layer epithelial cells were only partially destroyed in the distal part of the ciliary process.

In cyclocryotherapy, the ciliary process atrophied and lost its integrity. Some epithelial cells separated from the ciliary body in a balloon-like manner. With necrosis of the epithelial cells, the stroma of the ciliary process was also affected, resulting in hyperemia and edema. In addition, a large number of inflammatory cells infiltrated the ciliary body, causing capillary rupture and microbleeding.

**Figure 2:**
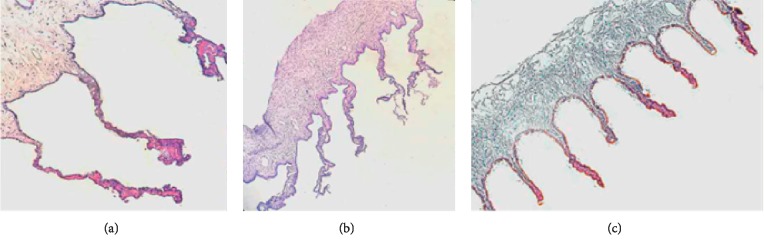
The difference among the UCP postoperative (a), cyclocryotherapy postoperative (b), and normal ciliary body (c). Magnification, ×40.

The historical examination revealed the differences in the ciliary body between the two groups of rabbit eyes, as shown in Figure 2.

### 3.6. MMP-1 Expression

**Figure 3:**
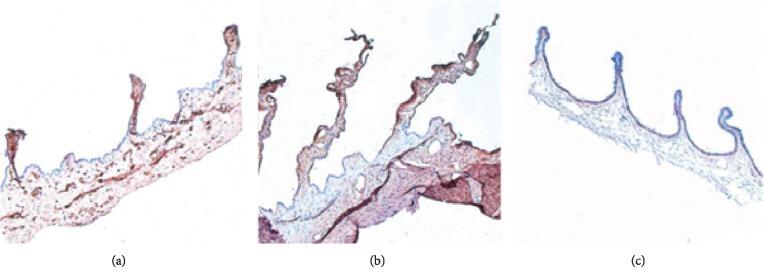
Distribution of MMP-1 in the UCP postoperative (a), cyclocryotherapy postoperative (b), and normal ciliary body (c). Magnification, ×40.

MMP-1 is a protein widely expressed in the ciliary body, especially in the ciliary process and ciliary muscle. MMP-1 not only can provide structural support for the cell but also reduce the resistance of the aqueous humor outflow pathway. Both UCP treatment and cyclocryotherapy aim to reduce the IOP by destroying the ciliary body. Therefore, the MMP-1 expression in the ciliary body after treatment can help to compare the damage to the ciliary body caused by two procedures, as shown in Figure 3.

## 4. Discussion

This study was designed to compare the efficacy and safety of UCP and cyclocryotherapy in NVG. During the six-month follow-up, we found that the two techniques had similar effect on reducing the IOP. Both obtained a high success rate with no peak or increase in IOP, which was a desirable result. This study showed that both traditional cyclocryotherapy and the newly developed UCP treatment could significantly reduce the IOP.

Although both operations are nonincision ring destruction and the effect of reducing IOP is similar, UCP is less invasive and destructive. The UCP treatment provided significant advantages in terms of pain relief and postoperative complications. No visual impairment occurred in the patients treated with UCP [12–14]. However, in the cyclocryotherapy group, visual impairment or loss of vision occurred in some cases. The number of patients with no vision increased from 9 to 18 after surgery. This result is probably related to the notable difference in temperature between the cryoprobe and the ciliary body during the surgery. As the temperature of the ciliary body is much higher than that at the tip of the probe and estimating the temperature difference accurately between the probe and the tissue is difficult, strict temperature control cannot be achieved [5, 15].

Furthermore, the degree of freezing is difficult to gauge, and the placement of the cryoprobe and the operator's technique may have an effect in this regard [16], for example, suboptimal intraoperative centering or moving of the device (which is positioned and maintained manually by the operator). Large contact areas of the cryoprobe may also induce the range that is larger than desired and cause excessive destruction by freezing the ciliary body and collateral tissues resulting in additional side effects such as a drop in visual acuity [17]. After performing a simple preoperative biometry eye examination, the appropriate UCP probe size is easily selected for each patient. The device is a custom-made ring-shaped probe containing miniaturized transducers and the ultrasound treatment is automatically implemented by the module. Thermal lesions generated by each transducer are precisely positioned in the ciliary body without damage to adjacent tissue which greatly improves the accuracy of treatment compared to cyclocryotherapy [14].

The number of complications in the patients treated by UCP was fewer than those in the patients treated by cyclocryotherapy. The UCP group had two cases of conjunctival hemorrhage, one case of corneal edema and two cases of hyphemia, which were all relieved one week after. However, the frequency of complications in cyclocryotherapy was relatively higher. Some patients developed serious inflammatory symptoms such as superficial punctate keratitis and iridocyclitis. This is reactive inflammation caused by freezing that destroys the vascular system of the ciliary body [18]. There were no related inflammatory reactions in the UCP group; the inflammation after UCP was better controlled compared with cyclocryotherapy. Six patients developed an anterior chamber flare, which indicates an increase in the aqueous humor proteins and fibrinous exudation caused by the damage to the blood-aqueous barrier in the frozen area [6]. Conversely, in the UCP treatment, the transducers in the circular probe can focus precisely on the targeted area without causing damage to the blood-aqueous barrier. The treatment probe can partially coagulate the ciliary body without the influence of the pigment. During the procedure, the ciliary body is heated up evenly and progressively to avoid the risk of tissue overheating and to protect the surrounding area [19]. Two cases of ocular hypotony and one case of atrophy of the eyeball occurred in both groups. After the withdrawal of antihypertensive medications, the IOP returned to normal and atrophy of the eyeball did not progress. This may be due to excessive pressure of surgeon exerted on the positioning cone or cryoprobe on the eye during the procedure with consequent deformation of the sclera and the ciliary body. One other reason could be that each patient has a different susceptibility to low temperature and ultrasound.

Moreover, a significant difference was also observed in pain relief. Although the two procedures could help alleviate the pain, pain relief was more rapid and significant in the UCP group. Pain was already greatly relieved on the first day after the UCP treatment, and no patient complained of pain in the later follow-up [20, 21]. Unfortunately, pain relief after cyclocryotherapy was insufficient, the effect was slow, and pain was significantly relieved one week after surgery. One patient underwent eye enucleation due to recurrent severe pain six months after the operation.

To better understand tissue damage caused by the UCP treatment and cyclocryotherapy, coronal sections of the ciliary body were observed after the two procedures in the animal experiment. The HE-stained sections showed an expansion of the stromal collagen fibers and blood vessels in the targeted area treated by the UCP. Heat coagulative necrosis was achieved in the ciliary body without completely destroying it. Except for the layers of pigmented and nonpigmented epithelial cells that produce aqueous humor, all the other parts remained the same with only slight scars [9, 22]. On the contrary, the sections in the cyclocryotherapy group showed that the ciliary processes were severely damaged and even separated from the ciliary body. Moreover, the epithelial cells disappeared and the stromal cells were damaged [15, 23, 24]. The affected area of cyclocryotherapy was relatively large, and limiting the targeted area to sub-millimeters such as the UCP treatment, which could bring more complications and increase the risk of surgery, was not possible. In further studies, through the distribution of MMP-1, we can observe the degree and range of damage to the ciliary body after both treatments. Clearly, the UCP treatment could locate the ciliary process more accurately, and the temperature design was more reliable. MMP-1 in double-layer epithelial cells was completely ablated, whereas MMP-1 in the other parts was preserved, thus proving a better protection of adjacent tissues. By contrast, MMP-1 in the ciliary process disappeared completely after cyclocryotherapy and was found only in the ciliary muscle, consistent with the results of the clinical follow-up [25, 26].

Based on clinical studies and animal experiments, UCP can be considered a new alternative to surgery for glaucoma that provides a gentler and more comfortable treatment than cyclocryotherapy. The specific probe design and the precise positioning improved the accuracy of the treatment. The UCP technique should be considered for patients with early-stage glaucoma, as indicated in the European literature [7, 27, 28]. More importantly, doctors can adapt the treatment dose to minimize functional impairment and patients' suffering. The UCP treatment is characterized by a short treatment duration, a good safety profile, and ease in application [29]. However, during the treatment procedure, the following key details still need to be considered. (1) The optical axis of the eye should be perpendicular to the horizontal line to ensure that the probe precisely targets the ciliary body for maximum effect. (2) The positioning cone should be placed in the center in accordance with the radius of the white scleral, which is essential for a successful treatment. (3) The positioning cone should always be filled with saline solution throughout the procedure for the transmission of the ultrasound beams [11].

In the UCP group, one patient experienced a failure of one sector because the surgeon released the pedal and interrupted the treatment by accident. In the future, this type of accident should be avoided.

In this controlled study, the mean IOP baseline of the enrolled patients was relatively high, and thus the treatment effect of the UCP in the NVG patients with a low baseline IOP still requires further investigation. The limitation of this study is also related to the small sample size. Studies with a large sample size or multicenter studies should be conducted, and follow-up time should be extended to observe the long-term effect of the two procedures.

In conclusion, the results showed that, compared with cyclocryotherapy, the UCP treatment has a better safety profile and a shorter recovery time. The UCP treatment can largely alleviate patients' suffering, thus making it an excellent alternative for the treatment of NVG. UCP offers a new treatment option to NVG patients before invasive surgeries.

## Figures and Tables

**Table 1:** Patients characteristics.

	UCP	Cyclocryotherapy	*P*
Patients	26	23	
Age, mean ± SD (range), year	60.1 ± 13.2 (25–80)	58.1 ± 17.4 (23–80)	0.984^a^
Sex			0.396^b^
Male	11	13	
Female	15	10	
Number of previous trabeculectomy			0.706^b^
*n* = 0	21	20	
*n* = 1	5	3	
*n* > 1	0	0	
Lens status			0.612^b^
Phakic	23	22	
Pseudophakic	3	1	
Aphakic	0	0	
IOP baseline, mean ± SD	54.6 ± 9.7	53.3 ± 11.7	0.389^a^
Preoperative hypotensive medications, mean ± SD	1.8 ± 0.8	1.9 ± 0.7	0.792^a^
Visual acuity, logMar			0.923^c^
Visual acuity	4	2	
Count fingers	1	1	
Hand motion	2	3	
Light perception	10	8	
No light perception	9	9	

^a^Mann–Whitney test, ^b^Fisher test, ^c^Chi-square test. IOP: intraocular pressure, SD: standard deviation.

**Table 2:** Intraocular pressure at baseline and during follow-up in the patients.

	UCP				Cyclocryotherapy				*P* value
	Mean ± SD IOP (no patients)	Relative IOP reduction (%)	Success rate (%)	*P* value compared with the baseline	Mean ± SD IOP (no patients)	Relative IOP reduction (%)	Success rate (%)	*P* value compared with the baseline	
Baseline	54.6 ± 9.7 (26)	NA	NA	NA	53.3 ± 11.7 (23)	NA	NA	NA	0.389^a^
Day 1	37.9 ± 11.1 (26)	30	73	0.000^d^	34.1 ± 8.6 (23)	34	70	0.000^d^	0.203^a^
Day 7	31.1 ± 10.4 (24)	42	83	0.000^d^	32.2 ± 9.8 (21)	37	81	0.000^d^	0.909^a^
Month 1	30.9 ± 11.4 (25)	43	88	0.000^d^	29.9 ± 10.9 (21)	43	76	0.000^d^	0.774^a^
Month 3	29.0 ± 10.7 (26)	45	84	0.000^d^	33.5 ± 11.1 (19)	36	79	0.000^d^	0.574^a^
Month 6	30.3 ± 9.4 (21)	42	76	0.000^d^	30.4 ± 9.1 (22)	40	77	0.000^d^	0.650^a^

^a^Mann–Whitney test, ^d^Wilcoxon test. NA, not applicable, IOP: intraocular pressure, SD: standard deviation.

**Table 3:** Pain assessment before operation and during follow-up in the patients.

	UCP	Cyclocryotherapy	*P* ^*∗*^
Preoperative	6.31 ± 1.8	6.35 ± 1.7	0.862
Operative night	4.08 ± 1.7	5.52 ± 1.6	0.004
Day 1	1.31 ± 1.4	4.61 ± 1.5	0.000
Day 7	0.46 ± 0.7	2.67 ± 1.9	0.000
Month 1	0.24 ± 0.6	1.57 ± 1.3	0.000
Month 3	0.08 ± 0.3	1.63 ± 1.3	0.000
Month 6	0.09 ± 0.3	1.09 ± 0.8	0.000

^*∗*^Mann–Whitney test.

**Table 4:** Comparison of visual acuity before and after cyclocryotherapy.

	0–0.1	Count fingers	Hand motion	Light perception	No light perception
Preoperative	2	1	3	8	9
Post-surgical	0	0	3	2	18

**Table 5:** Postoperative complications.

Ocular complications	UCP	Cyclocryotherapy
Conjunctival hyperemia	2	4
Subconjunctival hyperemia	0	1
Corneal edema	1	3
Superficial punctate keratitis	0	1
Hyphema	2	3
Aqueous flare	0	6
Anterior uveitis	0	1
Retinal detachment	0	1
Hypotony	2	2
Phthisis	1	1

## Data Availability

The data used to support the findings of this study are available from the corresponding author upon request.
